# Transplantation of Human Embryonic Stem Cell-Derived Retinal Cells into the Subretinal Space of a Non-Human Primate

**DOI:** 10.1167/tvst.6.3.4

**Published:** 2017-05-16

**Authors:** Jennifer R. Chao, Deepak A. Lamba, Todd R. Klesert, Anna La Torre, Akina Hoshino, Russell J. Taylor, Anu Jayabalu, Abbi L. Engel, Thomas H. Khuu, Ruikang K. Wang, Maureen Neitz, Jay Neitz, Thomas A. Reh

**Affiliations:** 1 Department of Ophthalmology, University of Washington, Seattle, WA, USA; 2 Buck Institute for Research on Aging, Novato, CA, USA; 3 Vitreoretinal Associates of Washington, Seattle, WA, USA; 4 Department of Biological Structure, University of Washington, Seattle, WA, USA; 5 Department of Cell Biology and Human Anatomy, University of California, Davis, CA, USA; 6 University of Wisconsin, Madison, WI, USA; 7 Universal Cells, Inc., Seattle, WA, USA; 8 Department of Bioengineering, University of Washington, Seattle, WA, USA

**Keywords:** stem cell transplantation, retina, primate, glaucoma, AMD

## Abstract

**Purpose:**

Previous studies have demonstrated the ability of retinal cells derived from human embryonic stem cells (hESCs) to survive, integrate into the host retina, and mediate light responses in murine mouse models. Our aim is to determine whether these cells can also survive and integrate into the retina of a nonhuman primate, *Saimiri sciureus,* following transplantation into the subretinal space.

**Methods:**

hESCs were differentiated toward retinal neuronal fates using our previously published technique and cultured for 60 to 70 days. Differentiated cells were further treated with 20 μM N-[N-(3,5-Difluorophenacetyl)-L-alanyl]-S-phenylglycine t-butyl ester (DAPT) for a period of 5 days immediately prior to subretinal transplantation. Differentiated cells were labeled with a lentivirus expressing GFP. One million cells (10,000 cells/μL) were injected into the submacular space into a squirrel monkey eye, using an ab externo technique.

**Results:**

RetCam imaging demonstrated the presence and survival of human donor cells 3 months after transplantation in the *S. sciureus* eye. Injected cells consolidated in the temporal macula. GFP^+^ axonal projections were observed to emanate from the central consolidation of cells at 1 month, with some projecting into the optic nerve by 3 months after transplantation.

**Conclusions:**

Human ES cell-derived retinal neurons injected into the submacular space of a squirrel monkey survive at least 3 months postinjection without immunosuppression. Some donor cells appeared to integrate into the host inner retina, and numerous donor axonal projections were noted throughout, with some projecting into the optic nerve.

**Translational Relevance:**

These data illustrate the feasibility of hESC-derived retinal cell replacement in the nonhuman primate eye.

## Introduction

Vision loss from diseases such as age-related macular degeneration and glaucoma, the leading causes of irreversible blindness in the developed world, results from degeneration of retinal cells, including photoreceptors and ganglion cells.^1,2^ At present, there are no effective means of restoring vision to patients with these diseases, although retinal cell replacement through transplantation has been investigated as a potential avenue to achieve this.^3–11^ Human embryonic stem cells (hESCs) and induced pluripotent stem cells (iPSCs) have been investigated in recent years as viable sources of retinal cells for transplantation.^5,6,10–12^ Early results from clinical trials utilizing subretinal transplantation of hESC-derived retinal pigmented epithelial (RPE) cells in patients with age-related macular degeneration (AMD) and Stargardt Macular Dystrophy have been reported.^13–15^ While RPE supplementation might slow retinal degenerative processes, it is likely that restoration of visual function will also ultimately require replacement of cells in the neurosensory retina.

While there are a number reports on embryonic stem cell-derived transplants of retinal cells in animal models,^3–11^ few studies have utilized human ESC-derived retinal cells for subretinal transplantation.^5,6,11^ A recent study reported the results of transplanted hESC-derived retinal sheets from a 3D culture system into two primate models of retinal degeneration.^10^ However, there remain several questions regarding the optimal configuration of donor cells (cell suspension versus sheets), barriers to integration and donor cell function, and the role of immunosuppression for transplantation.^11,16–19^ A recent report demonstrates the ability of transplanted murine neurons to form axons extending into the optic nerve head in the host mouse eye.^20^ A similar phenomenon has yet to be demonstrated in a host primate eye.

In this pilot study of a single nonhuman primate, we investigate the feasibility of subretinal transplantation of hESC-derived retinal neurons into a healthy retina that more closely resembles the human eye. We sought to determine the survival, integration, and ability of human donor cells to elaborate axons in a nonimmunosuppressed *Saimiri sciureus* eye.

## Methods

### Cell Culture and Retinal Induction

The H1 (WA01) hESC line was obtained from WiCell Research Institute. The cells were maintained in feeder-free conditions using TESR2 media (Stemcell Technologies, Vancouver, British Columbia, Canada) and Matrigel (BD Biosciences, Franklin Lakes, NJ). Retinal induction was performed as previously described. Briefly, embryoid bodies (EBs) were formed by treating undifferentiated hES colonies with dispase and type IV collagenase (Invitrogen, Grand Island, NY) and resuspended in approximately 150 100-cell clumps per milliliter in a six-well ultra-low attachment plate (VWR, Radnor, PA). These EBs were cultured for 3 days in the presence of mouse noggin (R&D Systems, Minneapolis, MN), human recombinant Dkk-1 (R&D Systems), and human recombinant insulin-like growth factor-1 (IGF-1; R&D Systems). On the fourth day, EBs were plated onto each poly-D-lysine-Matrigel (Collaborative Research, Inc., Bedford, MA) coated plates and cultured in the presence of DMEM/F12, B-27 supplement, N-2 Supplement (Invitrogen), mouse noggin, human recombinant Dkk-1, human recombinant IGF-1, and human recombinant basic fibroblast growth factor (bFGF; R&D Systems). The media was changed every 2 to 3 days for up to 3 weeks. The differentiated cells were maintained in media containing DMEM/F12, N2 supplement, B27 Supplement, NEAA, and penicillin-streptomycin antibiotic. Prior to transplantation, the cells were treated with Notch inhibitor, N-[N-(3,5-Difluorophenacetyl)-L-alanyl]-S-phenylglycine t-butyl ester (DAPT) (Sigma-Aldrich, St. Louis, MO) at 20-μM concentration for up to 5 days in the above described media.

One week prior to transplantation, differentiated cells were transduced with lentiviruses driving eGFP under the EF1α promoter as previously described.^5^ Cells were infected by overnight incubation with virus containing media. Cells were washed with phosphate buffered saline (PBS) next day and media replaced. The media was replaced at least 3 times over the next 7 days prior to transplantation.

### Virus Production and Infection

EF-1α-GFP lentivirus was generated using constructs provided by Charles Murry (University of Washington). Third-generation replication-incompetent lentivirus was made using the four-plasmid system. HEK-293 transfection was done using calcium phosphate precipitation and supernatant collected 48 to 72 hours later. The cleared supernatant was filtered through a 0.45-μm syringe filter, concentrated (Millipore Amicon filter, Millipore, Billerica, MA) aliquoted, and stored at −80°C until use.

### Real-Time Quantitative PCR (qPCR)

Total RNA was extracted from cultures using TriZol (Invitrogen) followed by chloroform extraction, DNase-1 (Qiagen, Waltham, MA) treatment followed by the Qiagen RNA mini cleanup kit. cDNA was reverse transcribed using Superscript II RT kit (Invitrogen) as per manufacturer's instructions. qPCR was performed for Hes5, Hes1, Pax6, Brn3b, and Recoverin using iTaq Universal Sybr Green (Bio-Rad) performed on the DNA Engine Opticon2 System (Bio-Rad, Hercules, CA) according to the protocol below: cycle 1: 95°C for 3 minutes, 1 repeat, cycle 2: 96°C for 10 seconds and 59°C for 60 seconds (data collection), 40 repeats; and results were normalized to β-actin levels. Results were normalized to β-actin levels. The following primer sequences were used: HES5-F: CTCAGCCCCAAAGAGAAAAA; HES5-R: GCTTAGCAGATCCTTGCTCCAT; HES1-F: ATGGAGAAAAATTCCTCGTCCC; HES1-R: TTCAGAGCATCCAAAATCAGTGT; PAX6-F: TCTAATCGAAGGGCCAAATG; PAX6-R: TGTGAGGGCTGTGTCTGTTC; BRN3B (POU4F2)-F: CTCGCTCGAAGCCTACTTTG; BRN3B (POU4F2)-R: GACGCGCACCACGTTTTTC; RCVRN-F: GCAGAGGTCCTATCCCATGA; RCVRN-R: AGTCATTGGAGGTGACATCG; β-actin-F: AGGCACCAGGGGCGTGAT; and β-actin-R: GCCCACATAGGAATCCTTCTGAC. All of the primers were designed for an amplicon length of between 70 and 170 base pairs.

### Subretinal Transplantation of Differentiated Cells

All animal procedures were approved by the Institutional Animal Care and Use Committee of the University of Washington and conducted in accordance with the ARVO Statement for the Use of Animals in Ophthalmic and Vision Research. The subretinal injection was performed by a vitreoretinal surgeon (T.R.K.) using a KDS model 210 syringe pump under a surgical microscope (Leica, Buffalo Grove, IL). A 0.5-cc luer lock syringe was connected to 30-gauge Teflon tubing (Hamilton 30TF double hub) with male luer lock adapters (Argon Med Devices, Plano, TX) at both ends, which was then connected to an iTRACK-275 microcatheter (iScience, Beaverton, OR). An optical fiber incorporated into the catheter for surgical illumination and guidance was connected to an iScience Interventional iLumin Fiberoptic Illuminator. All components were sterilized prior to use. The syringe and tubing were filled with sterile cell culture media to occupy the dead volume of approximately 180 μL. Immediately prior to injection, 300 μL differentiated retinal cells was withdrawn using the syringe pump at a rate of 100 μL/min.

The *S. sciureus* monkey was anesthetized using intramuscular injection of ketamine (15 mg/kg), intubated, and then maintained under gas anesthesia using sevoflurane. During the procedure, the monkey received 10 mg intramuscular (IM) ketoprofen and 25 mg intravenous (IV) cefazolin. The eye was dilated with two to three drops of 1% tropicamide and 1% atropine, and prepped with povidone iodine and draped in the usual sterile fashion. One corneal stay suture was placed, and the eye was rotated nasally. A temporal conjunctival peritomy was performed, and a radial sclerotomy was created 3 to 5 mm posterior to the limbus. A Sinskey hook was used to dissect the scleral fibers, and the Micro-Retractor (iScience Interventional/Ellex) was placed and sutured to the scleral surface. Using the ViscoDissection wiretip cannula to carefully inject Healon (Abbott Medical Optics, Abbott Park, IL) through the choroidal fibers, a small local anterior retinal detachment was created. The iTRACK-275 microcatheter was then inserted at a very low angle. With the aid of a macular viewing lens, the catheter was then guided through the subretinal space until the lighted tip was advanced into the macula. The subretinal injection was then performed, injecting 100 μL volume at a concentration of 10,000 retinal cells/μL. The maximum diameter of the ball end tip of the iTRACK-275 was 275 μm, and the shaft was 200 μm in outer diameter and 150 μm in inner diameter. This cannula diameter and speed of delivery did not adversely affect cell viability (data not shown). A macular retinal detachment was noted without obvious reflux at the anterior catheter insertion site. The catheter was carefully removed, and the scleral incision and conjunctiva were closed with interrupted 7-0 Vicryl sutures. Subconjunctival injections of 50 μL cefazolin (100mg/mL) and 50 μL decadron (10 mg/mL) were given.

### Fundus Imaging

Fundus images were obtained using the RetCam II (Massie Laboratories, Pleasanton, CA). The primate was anesthetized for all exams, and the eye was dilated with two to three drops of 1% tropicamide and 1% atropine. Goniosol (hydroxypropyl methylcellulose) was placed between the RetCam lens and the corneal surface. Standard light conditions were used for color fundus images, and images for GFP visualization were taken under conditions used for fluorescein angiography. Indirect ophthalmoscopy with a 28-diopter lens was also performed at each time point.

### Immunocytochemistry and Immunohistochemistry

Cells were fixed with 4% paraformaldehyde for 30 minutes. The eye was enucleated 2 months after surgery and processed for histology. The eye was fixed with 4% paraformaldehyde overnight. The anterior segment (cornea/lens) was removed and the eye cup incubated in a solution of 1:1 mixture of 30% sucrose and O.C.T (Tissue-TEK) at 4°C overnight. The eye cup was then embedded in O.C.T. and 25- to 30-μm transverse sections were cut, placed on Superfrost Plus glass slides, and stored at −80°C.

Cells and tissues sections were permeabilized with Triton-X100 and blocked with 5% donkey serum. Primary antibody staining was carried out overnight at 4°C. Primary antibodies used include GFP (1:250, Santa Cruz and Acris, Atlanta, GA) Calbindin (D-28K; 1:1000, Swant, Marly, Switzerland), Ki-67 (1:250, Abcam, Eugene, OR), PH3 (1:500, Novus), NeuN1 (1:200, Millipore), TUJI (1:1000, Covance, Princeton, NJ), STEM121 (1:500, Takara/Clontech, Mountain View, CA), HuC/D (1:100, Invitrogen), S-Opsin (1:250, Santa Cruz), Recoverin (1:1000–1:2000, Chemicon, Billerica, MA), and OTX2 (1:200, R&D Systems). Secondary antibody staining was performed using the corresponding Alexa-488, Alexa-568, and Alexa-647 fluorescent-tagged antibodies (1:250–500; Molecular Probes, Grand Island, NY) and nuclei stained with 4′,6-Diamidine-2′-phenylindole dihydrochloride (DAPI; Sigma-Aldrich) for 1 hour at room temperature, and cover-slipped with Fluoromount-G with DAPI. Images were taken using a Zeiss confocal microscope.

## Results

### Enhanced Differentiation of Retinal Progenitor Cells from hESCs into Retinal Neurons with Notch Inhibition

**Figure 1:**
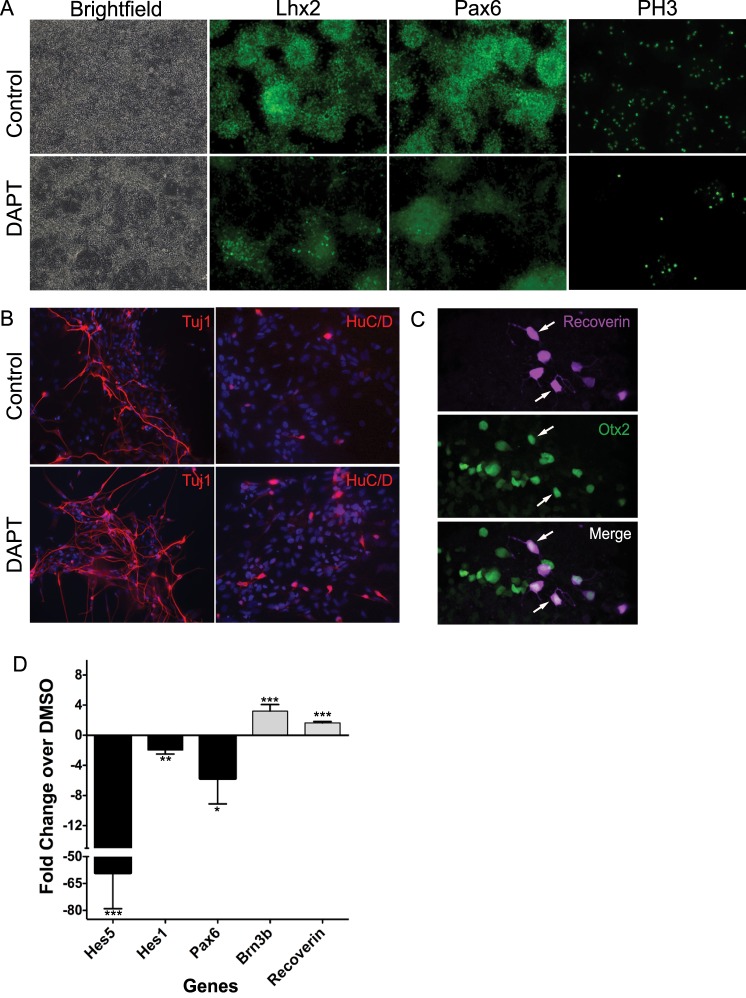
DAPT induces retinal progenitor differentiation and inhibits proliferation. hESCs were differentiated toward retinal neuronal fates. (A) Brightfield and immunostaining of control and DAPT-treated cells after 5 days. A decrease in the number of rosettes, LHX2 and PAX6 expression, along with decreased PH3 staining of mitotic cells was observed in DAPT-treated cells. (B) DAPT treatment resulted in increased TUJ1- and HuC/D-expressing retinal cells. (C) Immunostaining reveals retinal neurons expressing photoreceptor markers, recoverin, and OTX2 (*arrows*). (D) RT-PCR analysis demonstrated decreased *PAX6* and increased *BRN3B* and *RCVRN* expression in DAPT-treated cells compared to DMSO controls. Mean ± SEM, *P < 0.05, **P < 0.01, ***P < 0.005.

The H1 (WA01, WiCell Research Institute) hESC line was differentiated into retinal progenitor cells (RPCs) as described previously.^21^ After 60 to 70 days of differentiation, 20 μM of DAPT was added to the culture for 5 days prior to the subretinal injection. Inhibition of Notch signaling by the addition of the γ-secretase inhibitor, DAPT, resulted in the flattening of neural rosettes along with decreased expression of retinal progenitor markers *LHX2*, *PAX6*, *HES5*, and *HES1* as detected by immunofluorescence staining and qPCR (Figs. 1A, 1D). There was a concomitant increase in the expression of TUJ1 (inner retinal neurons and cell processes), HU C/D (amacrine and RGCs), BRN3B (RGCs), and RCVRN (photoreceptors) expression (Figs. 1B, 1D). Immunofluorescence staining also revealed retinal cells expressing both RCVRN and OTX2 (Fig. 1C, arrows). Confocal microscopy images of differentiated retinal cells prior to injection were quantified for expression of RCVRN and OTX2, which demonstrated OTX2^+^ cells comprising 16.0% ± 4% (mean% ± SEM) of the total DAPI^+^ cells, and Recoverin^+^ cells comprising 3.7% ± 5%. Consistent with previously published results,^22^ there were a decreased number of mitotic cells as detected by phosphohistone 3 (PH3) staining (Fig. 1A).

### GFP^+^ hESC-Derived Cells Are Identified in the Subretinal Space

**Figure 2:**
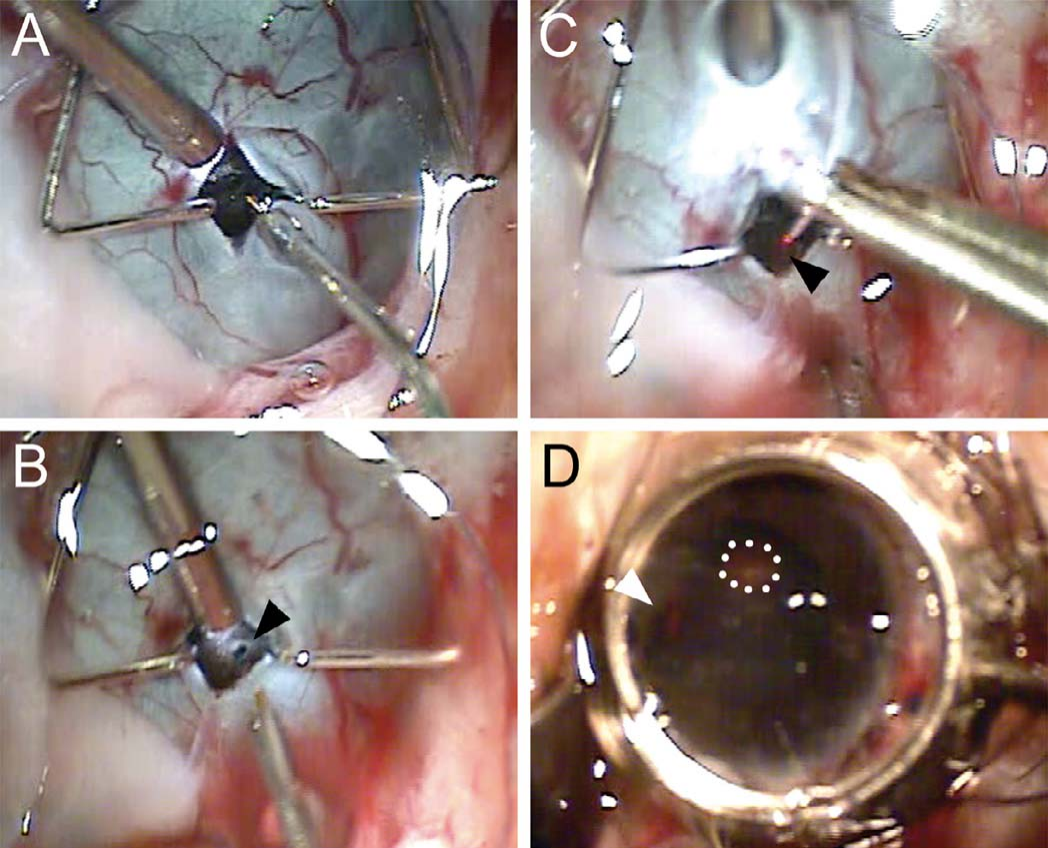
Ab externo delivery of hESC-derived retinal cells into the subretinal space of the *Saimiri sciureus* eye. (A) A radial sclerotomy was created and the incision was spread with a microretractor. (B) Dissection of choroid fibers and a choroidotomy was created using a wire-tipped cannula (*arrowhead*). A small amount of Healon was infused through the choroidotomy to create a subretinal bleb. (C) The subretinal catheter with a lighted fiberoptic tip (*arrowhead*) was inserted at a low angle into the subretinal space. (D) The catheter was advanced along the subretinal space into the superior macula, and the lighted (*red*) catheter tip could be visualized through a contact lens (*arrowhead*). Optic nerve (outlined by *dotted line*, D). The subretinal injection was performed under direct visualization.

**Figure 3:**
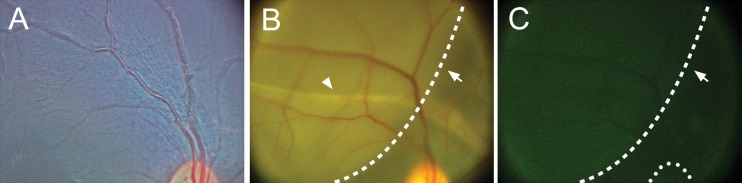
hESC-derived retinal cells transplanted into the superior macula. One million cells in a volume of 100 μL (10,000 cells/μL) were injected into the subretinal space of the superior macula. (A) Preinjection image of the superior macula and optic nerve. Donor cells were transduced with a lentivirus expressing EF1α-GFP. (B) Immediately postinjection, a localized subretinal detachment at the subretinal cannula infusion extending from the superior arcade into the central macula (*dotted line*, *arrow*). No retinal holes or tears were identified. The subretinal catheter track of RPE hypopigmentation could be appreciated (*arrowhead*). (C) GFP^+^-injected cells could be visualized in the subretinal space with RetCam imaging (*dotted line*, *arrow*). Optic nerve is delineated (*dotted line*).

One week prior to transplantation, hESC differentiated cells were transduced with lentiviruses expressing eGFP under control of the EF1α promoter. GFP^+^ hESC-derived retinal cells were harvested, and 1,000,000 cells in a 100-μL volume were introduced into the subretinal space. The subretinal injection was targeted to the superior macula using a lighted subretinal catheter via an ab externo technique (Fig. 2). Color fundus images taken immediately postoperatively show a shallow subretinal fluid bleb in the macula (Fig. 3). Additional imaging revealed GFP fluorescence in the corresponding area of the subretinal fluid bleb, indicating the presence of GFP^+^ cells in the subretinal space. A subretinal catheter track, presumably made by disruption of the RPE, was noted immediately postoperatively and was not observed at subsequent time points. There was likely some damage to the outer retina along the subretinal track, although no gross injury (e.g., retinal tear) to the neural retina at the time of surgery or postoperatively was observed. Post mortem histology did not reveal obvious retinal changes in the vicinity of the catheter track; however, specific histologic study of the catheter track was not performed. Upon examination with indirect ophthalmoscopy, the retina and RPE outside of the injection site and catheter track were normal, and no retinal tears or holes were noted in either the macula or periphery.

### Survival of hESC-Derived RPCs in the Subretinal Space and Extension of Axonal Projections

**Figure 4:**
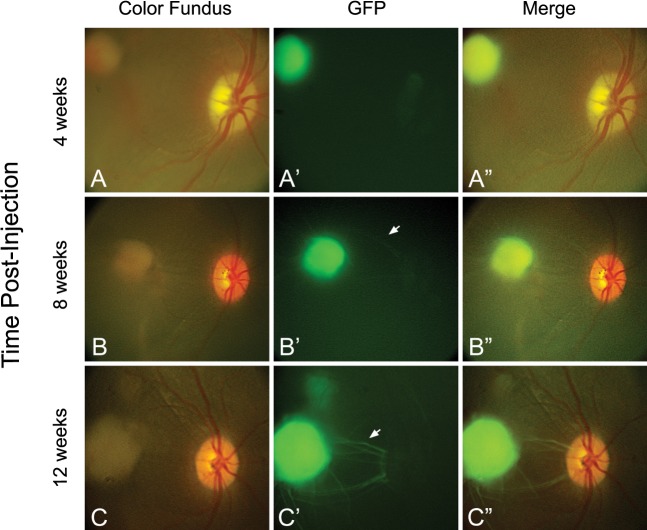
Transplanted hESC-derived retinal cells survive and extend axonal projections. (A) At 4 weeks, the injected hESC-derived retinal cells were observed to have consolidated in the posterior pole temporal to the fovea, corresponding to the most inferior and posterior extent of the original subretinal injection. (B) At 8 weeks postinjection, axonal projections emanating from the subretinal cell mass were observed, with some extending toward the optic nerve (*arrow*). (C) Additional projections were noted 12 weeks postinjection (*arrow*), with some projections appearing to change direction to travel along arcuate nerve fiber bundles with the host retinal NFL. Despite the absence of immunosuppression, there was no evidence of anterior or posterior segment inflammation at any time posttransplantation.

At 4 weeks after the subretinal injection, a subretinal consolidation of GFP fluorescent cells was noted in the macula at the most inferior extent of the original subretinal bleb (Fig. 4A-A”). There appeared to be very shallow subretinal fluid surrounding the cell aggregation on examination by indirect ophthalmoscopy, although the fluid appeared to be resolved by 8 weeks postinjection. No retinal tears or holes were noted at any of the time points. At 8 weeks postinjection, we observed axonal projections emanating from the aggregation, with some extending toward the optic nerve (Fig. 4B-B”, arrow). Additional and more robust projections were noted 3 months postinjection (Fig. 4C-C”). Although the axonal projections emanated in all directions from the cell aggregation at the early time points, at 3 months, increasing number of GFP^+^ axons followed the route of the typical anatomy of the nerve fiber layer (NFL) toward the optic nerve (Fig. 4C', arrow).

Although it was not possible to precisely measure the size of the subretinal aggregate over the 12-week time period in the absence of longitudinal optical coherence tomography (OCT) imaging, there did appear to be a slight increase in the size of the fluorescent subretinal aggregate relative to the optic nerve over the 12-week time period in the RetCam images. This could either be due to some limited proliferation of the cells early after subretinal transplantation or tangential spread of cells from the subretinal cell aggregate over time. There was no evidence of gross anterior segment inflammation or vitritis by indirect ophthalmoscopy at postinjection time points.

### Transplanted Cells Migrate into Inner Retinal Cell Layers and Extend Axonal Projections into the Optic Nerve

**Figure 5:**
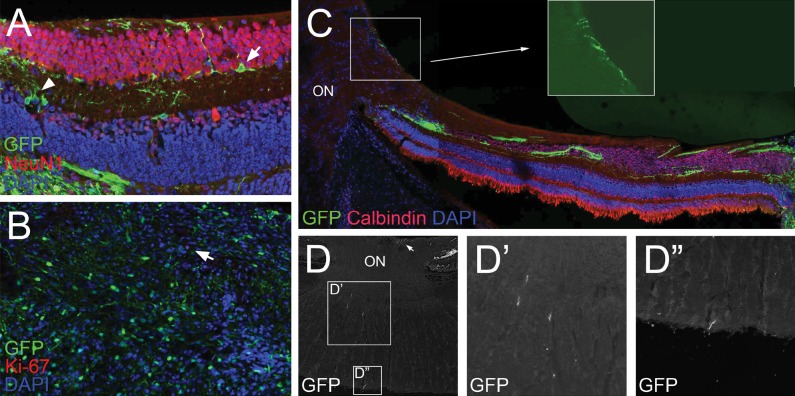
Transplanted cells migrate into inner retinal cell layers and extend axonal projections into the optic nerve. (A) At 12 weeks postinjection, few GFP^+^ cells were observed in the inner nuclear layer (*arrowhead*) and GCL (*arrow*). (B) Immunostaining of the subretinal mass of nonintegrated cells revealed few Ki-67^+^ cells (*arrow*). (C) Axonal projections emanating from the transplanted cells extended along the inner plexiform layer, GCL, and NFL. GFP^+^ projections were noted entering at the optic nerve head (D, *inset* and D, *arrow*), and extending into the retrolaminar optic nerve (D-D”). ON, optic nerve.

Histology of the retina 3 months postinjection showed some GFP^+^ cells located in the ganglion cell layer (GCL; Fig. 5A, arrow) and inner nuclear layer (Fig. 5A, arrowhead). The subretinal aggregate of cells comprised cells with varying levels of GFP expression, and immunostaining of the subretinal aggregate revealed few rare Ki-67^+^ cells (Fig. 5B, arrow). Similar results were obtained with PH3 staining (data not shown). In the host retina, axonal projections detected by GFP fluorescence were noted to extend along the inner plexiform layer, NFL, and to a lesser extent, the GCL (Figs. 5A, 5C). Interestingly, GFP fluorescence in axonal projections were noted at the optic nerve head (Fig. 5C, inset and 5D, arrow) and were detected at the most distal extent of the retrolaminar optic nerve of the enucleated globe (∼1.5 mm; Fig. 5D-D”).

**Figure 6:**
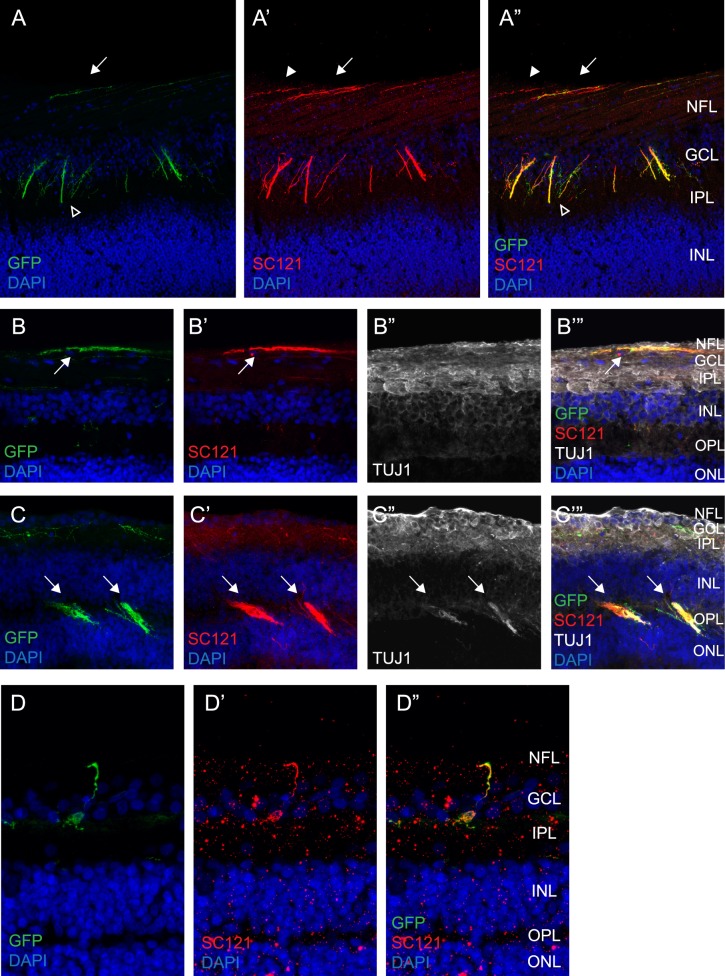
GFP^+^ cells and axonal extensions co-label with the human specific cell marker, STEM121. (A) Immunostaining at 12 weeks postinjection reveals GFP^+^ axons in the host NFL (*arrow*), as well as nonanatomical axonal projections through the inner plexiform layer and GCL (*open arrowhead*). GFP^+^ axonal extensions co-localize with STEM121 (human specific marker) immunostaining (A’ and A”, *arrow*), but some STEM121^+^ axonal extensions do not co-localize with the GFP signal (A’ and A”, *closed arrowhead*). (B-B’”) GFP^+^/STEM121^+^ axons are observed in the host NFL, which is strongly labeled with TUJ1 (*arrow*). (C-C’”) TUJ1 co-labeling of donor GFP^+^/STEM121^+^ axons (*arrows*) is evident in the outer plexiform layer where there are comparatively lower levels of TUJ1 expression in the host retina. (D-D”) A few GFP^+^ cells that co-label with STEM121 are located in the GCL and extend axonal projections into the NFL of the host retina. INL, inner nuclear layer; IPL, inner plexiform layer; ONL, outer nuclear layer; OPL, outer plexiform layer.

GFP^+^ cells and axonal extensions were examined for expression of a human-specific cytoplasmic marker, STEM121.^10^ GFP^+^ cells and axonal extensions co-labeled with the human marker; however, we observed that some human marker positive axons were not GFP^+^ (Fig. 6A, closed arrowhead and arrow). This finding is consistent with our method of lentiviral-mediated transduction of GFP, where the majority but not every donor cell was labeled with GFP prior to subretinal transplantation. Some of the GFP^+^ axonal projections extended along the host NFL (Figs. 6A, 6B), although some GFP^+^ axonal extensions clearly did not conform to the normal host anatomy (Fig. 6A, open arrowhead, and 6C, arrows). TUJ1 staining in the *S. sciureus* host retina was most prominent in the optic nerve, NFL, and GCL, with decreasing signal through the inner nuclear and outer plexiform layers (Figs. 6B”, 6C”). Co-expression of GFP^+^/STEM121^+^ axons with TUJ1 was evident in the NFL (Figs. 6B'–B'”), although TUJ1 labeling of the GFP^+^/STEM121^+^ donor axons was most evident in the OPL where there was comparatively lower levels of TUJ1 immunolabeling in the host retina (Figs. 6C–C'”). We found occasional GFP^+^ cells, co-labeled with the human-specific marker, located in the host GCL with axonal projections along the NFL (Figs. 6D–D”). Additional retinal markers were examined by immunohistochemistry for co-labeling with GFP^+^ cells. HU C/D weakly co-labeled with some GFP^+^ donor cells, but there was no obvious co-labeling with OTX2, RCVRN, or S-Opsin (data not shown).

## Discussion

This study demonstrates that hESC-derived retinal neurons injected into the subretinal space of a squirrel monkey (*S. sciureus*) eye have the potential to survive at least 3 months. Transplanted cells migrated into the inner nuclear layers, and in vivo imaging revealed the time course of axonal projections extending into the host retina, optic nerve head, and retrolaminar optic nerve.

The hESC-retinal cells used in this study were differentiated for 70 to 80 days in culture prior to transplantation, as previously published.^5,21^ Nelson et al.^23^ first demonstrated that exposure to the γ-secretase inhibitor, DAPT, at an early RPC stage causes a coordinated differentiation resulting in the production of developmental stage-appropriate retinal cell types. DAPT treatment also results in a decrease in the number of mitotic cells and an increase in Crx^+^ photoreceptor precursors and ganglion cells.^22,24,25^ Consistent with this, we found that DAPT exposure for a period of 5 days, immediately prior to subretinal transplantation, led to a decrease in the number of mitotic RPCs and increased expression of ganglion cell and photoreceptor markers.

We observed vertical migration of some transplanted cells from the subretinal space into inner layers of the retina. Notably, this occurred in a nondiseased primate retina with intact outer retinal layers and outer limiting membrane. Most previous transplantation studies into rodent models, including those published by our lab, and those utilizing a damaged primate retina substrate, have not observed significant integration into the inner retina. The observation of vertical integration through the retina in our study may be the result of species differences in the barriers to cellular integration, cell preparation (suspension versus sheets), or intact versus damaged primate retina.

Prior attempts at transplanting human Müller-derived retinal ganglion cells (RGCs) either alone or on scaffolds onto the inner retinal surface demonstrated their ability to integrate into the GCL of immune-suppressed rat and feline models of RGC depletion but did not demonstrate any ability to form axonal extensions.^26,27^ Further, while hESC-derived retinal cells injected into the epiretinal space in another study demonstrated limited integration into the GCL, no axonal projections were observed into the optic nerve.^11^ In the only other primate transplantation study to use hESC-derived cells, the grafted cells demonstrated the ability to integrate into the outer retinal layers in the damaged primate eye, but again there were no observable axonal projections through the retina.^10^ Recently, primary murine RGCs were shown to have the ability to integrate and extend processes into the optic nerve and lateral geniculate nucleus after intravitreal injection into the murine eye.^20^ Similar to these findings in the murine eye, small GFP^+^ axonal projections from hESC-derived donor cells were noted on initial fundus photography at 1 month postinjection in our primate model. At later time points, the GFP^+^ projections extended further radially from the subretinal cell mass and some altered course to extend along arcuate nerve fiber bundles within the host retinal NFL toward the optic disc. A subset of GFP^+^ projections extended into the optic disc in the inner circumference of the neuroretinal rim of the optic disc, consistent with the pattern of host axonal projections originating form the central macula.^28^ GFP^+^ axons were detected at approximately 2 mm beyond the lamina cribrosa, the most distal extent of the optic nerve available for study on histopathology in our study animal. The ability of hESC-derived cells to project extensions through various retinal layers, including the NFL, and into the optic nerve suggests that donor cells may have the ability to integrate into host retinal and central nervous system circuitry. If so, replacement of RGCs lost in diseases such as glaucoma may ultimately be possible.

Recent studies have highlighted the importance of major histocompatibility complex (MHC) matching and immune suppression to the success of RPE and photoreceptor survival after transplantation in murine models.^8,19,29–31^ Subretinal transplantation of iPSC-derived RPE allografts into MHC-matched adult cynomolgus monkeys without immunosuppression did not result in any signs of rejection, whereas MHC-mismatched recipients demonstrated signs of inflammation and retinal tissue damage around graft sites.^32^ Photoreceptor survival was significantly improved with suppression of the T cell-mediated immune response in murine models at 3 months posttransplantation.^8^ A more recent study demonstrates that photoreceptor integration is improved in IL2rγ-deficient mice compared to immunocompetent controls, with functional visual restoration persisting up to 9 months posttransplantation.^19^ Despite the integration of some retinal cells into the host retina in our study, we did not observe integration of any photoreceptor cells into the host retina or in the subretinal space at 12 weeks posttransplantation. This may be due to the relatively few photoreceptors present in the donor cell population; however, based on recent studies, it is also possible that the lack of immune suppression in the recipient inhibited photoreceptor cell survival. While we did not detect any physical exam findings of inflammation posttransplantation, immunohistochemical staining for host immune markers in the retina was limited by the scarcity of available *S. sciureus* primary antibodies.

Donor cells were transduced with lentiviruses expressing eGFP in order to identify them in posttransplantation studies. The lentivirus-containing media was removed from the donor cells and changed multiple times in the week prior to the subretinal injection surgery. While it is possible that residual lentiviruses could have labeled host retinal cells,^33^ this is less likely due to our observation that all of the GFP^+^ cells we observed were also STEM121^+^ (human-specific cytoplasmic marker). We did observe, however, that not all STEM121^+^ cells were GFP^+^. This is likely due to limitations in lentiviral transduction efficiency. Separately, recent studies have reported evidence of material transfer from transplanted donor photoreceptors to recipient retinas.^34–36^ Both GFP and the enzyme Cre recombinase were reported to transfer from donor to host photoreceptors in murine retinas. Because STEM121 is also a cytoplasmic protein, we cannot completely rule out the role of material transfer to the host retina in our study. However, it is notable that many of the GFP^+^/STEM121^+^ cells found posttransplantation extended axons both in the host NFL, as well as nonanatomical projections through the inner plexiform layer and GCL, making it more likely that these were from donor rather than host cells.

We chose to use an ab externo surgical technique in order to minimize both intraocular surgical manipulation with a pars plana vitrectomy and to minimize the reflux of transplanted cells into the vitreous cavity through the retinotomy that is observed in larger eyes after subretinal injections.^37,38^ The lighted fiberoptic tip allowed for targeting of the area for the subretinal injection, and the submacular detachment was achieved without any obvious trauma to the overlying retina. We also did not observe any obvious reflux at the choroidal entry site. A subretinal track as evidenced by a hypopigmented line through the RPE was noted from the periphery to the targeted retina after the procedure; however, the track was not observed on fundus exam at 1 month after the injection. Finally, there was no evidence of cataract formation, intraretinal hemorrhage, vitreous hemorrhage, rhegmatogenous retinal detachment, or proliferative vitreoretinopathy at any point after the procedure. While this surgical approach can be considered in human patients, our transplanted cells appeared to consolidate at the most inferior extent of the localized retinal detachment in a gravity-dependent manner. Future studies will need to account for this either by decreasing the total number of donor cells or restricting positioning during the postoperative period prior to resolution of the subretinal fluid. An alternative to avoid this is transplantation of retinal sheets,^10^ although this appears to require a substantially more invasive procedure involving a pars plana vitrectomy, retinal incision, air/fluid exchange, and silicone oil placement.

To date, two nonhuman primate studies involving subretinal injections of human stem cell derived cells have been reported.^10,37^ In our pilot experiment, human ES cell-derived retinal neurons injected into the subretinal space of a squirrel monkey (*S. sciureus*) survived at least 3 months postinjection without immunosuppression. While there was no obvious photoreceptor integration, in vivo imaging revealed a time course whereby donor cells extended axonal projections into the host retina and optic nerve over a period of 3 months. Further questions to be answered include the best surgical approach, donor cell configuration and dose, and need for immunosuppression. Primate eyes have the advantage of being similar in structure to the human eye, including a macula and fovea. Both prior studies used either topical or systemic immunosuppression, but our study indicates that immunosuppression may not be necessary for the survival of donor cells in the subretinal space.

## References

[1] Ambati J, Fowler BJ. Mechanisms of age-related macular degeneration. *Neuron*. 2012; 75: 26–39. 22794258 10.1016/j.neuron.2012.06.018 PMC3404137

[2] Klein R, Chou CF, Klein BE, Zhang X, Meuer SM, Saaddine JB. Prevalence of age-related macular degeneration in the US population. *Arch Ophthalmol*. 2011; 129: 75–80. 21220632 10.1001/archophthalmol.2010.318

[3] Banin E, Obolensky A, Idelson M, Retinal incorporation and differentiation of neural precursors derived from human embryonic stem cells. *Stem Cells*. 2006; 24: 246–257. 16123388 10.1634/stemcells.2005-0009

[4] Gonzalez-Cordero A, West EL, Pearson RA, Photoreceptor precursors derived from three-dimensional embryonic stem cell cultures integrate and mature within adult degenerate retina. *Nat Biotechnol*. 2013; 31: 741–747. 23873086 10.1038/nbt.2643 PMC3826328

[5] Lamba DA, Gust J, Reh TA. Transplantation of human embryonic stem cell-derived photoreceptors restores some visual function in Crx-deficient mice. *Cell Stem Cell*. 2009; 4: 73–79. 19128794 10.1016/j.stem.2008.10.015 PMC2713676

[6] Lamba DA, McUsic A, Hirata RK, Wang PR, Russell D, Reh TA. Generation, purification and transplantation of photoreceptors derived from human induced pluripotent stem cells. *PLoS One*. 2010; 5: e8763. 20098701 10.1371/journal.pone.0008763 PMC2808350

[7] Tucker BA, Park IH, Qi SD, Transplantation of adult mouse iPS cell-derived photoreceptor precursors restores retinal structure and function in degenerative mice. *PLoS One*. 2011; 6: e18992. 21559507 10.1371/journal.pone.0018992 PMC3084746

[8] West EL, Pearson RA, Barker SE, Long-term survival of photoreceptors transplanted into the adult murine neural retina requires immune modulation. *Stem Cells*. 2010; 28: 1997–2007. 20857496 10.1002/stem.520 PMC3272388

[9] Zhou L, Wang W, Liu Y, Differentiation of induced pluripotent stem cells of swine into rod photoreceptors and their integration into the retina. *Stem Cells*. 2011; 29: 972–980. 21491544 10.1002/stem.637 PMC4263955

[10] Shirai H, Mandai M, Matsushita K, Transplantation of human embryonic stem cell-derived retinal tissue in two primate models of retinal degeneration. *Proc Natl Acad Sci U S A*. 2016; 113: E81–E90. 26699487 10.1073/pnas.1512590113 PMC4711854

[11] Hambright D, Park KY, Brooks M, McKay R, Swaroop A, Nasonkin IO. Long-term survival and differentiation of retinal neurons derived from human embryonic stem cell lines in un-immunosuppressed mouse retina. *Mol Vis*. 2012; 18: 920–936. 22539871 PMC3335781

[12] Nazari H, Zhang L, Zhu D, Stem cell based therapies for age-related macular degeneration: the promises and the challenges. *Prog Retin Eye Res*. 2015; 48: 1–39. 26113213 10.1016/j.preteyeres.2015.06.004

[13] Schwartz SD, Hubschman JP, Heilwell G, Embryonic stem cell trials for macular degeneration: a preliminary report. *Lancet*. 2012; 379: 713–720. 22281388 10.1016/S0140-6736(12)60028-2

[14] Schwartz SD, Regillo CD, Lam BL, Human embryonic stem cell-derived retinal pigment epithelium in patients with age-related macular degeneration and Stargardt's macular dystrophy: follow-up of two open-label phase 1/2 studies. *Lancet*. 2015; 385: 509–516. 25458728 10.1016/S0140-6736(14)61376-3

[15] Schwartz SD, Tan G, Hosseini H, Nagiel A. Subretinal transplantation of embryonic stem cell-derived retinal pigment epithelium for the treatment of macular degeneration: an assessment at 4 years. *Invest Ophthalmol Vis Sci*. 2016; 57: ORSFc 1–9. 27116660 10.1167/iovs.15-18681

[16] Enzmann V, Faude F, Wiedemann P, Kohen L. Immunological problems of transplantation into the subretinal space. *Acta Anat (Basel)*. 1998; 162: 178–183. 9831766 10.1159/000046484

[17] Xian B, Huang B. The immune response of stem cells in subretinal transplantation. *Stem Cell Res Ther*. 2015; 6: 161. 26364954 10.1186/s13287-015-0167-1 PMC4568575

[18] Neves J, Zhu J, Sousa-Victor P, Immune modulation by MANF promotes tissue repair and regenerative success in the retina. *Science*. 2016; 353 :aaf3646. 10.1126/science.aaf3646 PMC5270511 27365452

[19] Zhu J, Cifuentes H, Reynolds J, Lamba DA. Immunosuppression via loss of IL2rγ enhances long-term functional integration of hESC-derived photoreceptors in the mouse retina. *Cell Stem Cell*. 2017; 20: 374–384. 28089909 10.1016/j.stem.2016.11.019

[20] Venugopalan P, Wang Y, Nguyen T, Huang A, Muller KJ, Goldberg JL. Transplanted neurons integrate into adult retinas and respond to light. *Nat Commun*. 2016; 7: 10472. 26843334 10.1038/ncomms10472 PMC4742891

[21] Lamba DA, Karl MO, Ware CB, Reh TA. Efficient generation of retinal progenitor cells from human embryonic stem cells. *Proc Natl Acad Sci U S A*. 2006; 103: 12769–12774. 16908856 10.1073/pnas.0601990103 PMC1568922

[22] Osakada F, Ikeda H, Mandai M, Toward the generation of rod and cone photoreceptors from mouse, monkey and human embryonic stem cells. *Nat Biotechnol*. 2008; 26: 215–224. 18246062 10.1038/nbt1384

[23] Nelson BR, Hartman BH, Georgi SA, Lan MS, Reh TA. Transient inactivation of Notch signaling synchronizes differentiation of neural progenitor cells. *Dev Biol*. 2007; 304: 479–498. 17280659 10.1016/j.ydbio.2007.01.001 PMC1979095

[24] Nelson BR, Gumuscu B, Hartman BH, Reh TA. Notch activity is downregulated just prior to retinal ganglion cell differentiation. *Dev Neurosci*. 2006; 28: 128–141. 16508310 10.1159/000090759

[25] Riazifar H, Jia Y, Chen J, Lynch G, Huang T. Chemically induced specification of retinal ganglion cells from human embryonic and induced pluripotent stem cells. *Stem Cells Transl Med*. 2014; 3: 424–432. 24493857 10.5966/sctm.2013-0147 PMC3973714

[26] Becker S, Eastlake K, Jayaram H, Allogeneic transplantation of Müller-derived retinal ganglion cells improves retinal function in a feline model of ganglion cell depletion. *Stem Cells Transl Med*. 2016; 5: 192–205. 26718648 10.5966/sctm.2015-0125 PMC4729554

[27] Singhal S, Bhatia B, Jayaram H, Human Müller glia with stem cell characteristics differentiate into retinal ganglion cell (RGC) precursors in vitro and partially restore RGC function in vivo following transplantation. *Stem Cells Transl Med*. 2012; 1: 188–199. 23197778 10.5966/sctm.2011-0005 PMC3659849

[28] Jonas JB, Dichtl A. Evaluation of the retinal nerve fiber layer. *Surv Ophthalmol*. 1996; 40: 369–378. 8779083 10.1016/s0039-6257(96)80065-8

[29] Lu B, Wang S, Girman S, McGill T, Ragaglia V, Lund R. Human adult bone marrow-derived somatic cells rescue vision in a rodent model of retinal degeneration. *Exp Eye Res*. 2010; 91: 449–455. 20603115 10.1016/j.exer.2010.06.024

[30] Sohn EH, Jiao C, Kaalberg E, Allogenic iPSC-derived RPE cell transplants induce immune response in pigs: a pilot study. *Sci Rep*. 2015; 5: 11791. 26138532 10.1038/srep11791 PMC4490339

[31] Zhao T, Zhang ZN, Westenskow PD, Humanized mice reveal differential immunogenicity of cells derived from autologous induced pluripotent stem cells. *Cell Stem Cell*. 2015; 17: 353–359. 26299572 10.1016/j.stem.2015.07.021 PMC9721102

[32] Sugita S, Iwasaki Y, Makabe K, Successful transplantation of retinal pigment epithelial cells from MHC homozygote iPSCs in MHC-matched models. *Stem Cell Reports*. 2016; 7: 635–648. 27641649 10.1016/j.stemcr.2016.08.010 PMC5063629

[33] West EL, Gonzalez-Cordero A, Hippert C, Defining the integration capacity of embryonic stem cell-derived photoreceptor precursors. *Stem Cells*. 2012; 30: 1424–1435. 22570183 10.1002/stem.1123 PMC3580313

[34] Pearson RA, Gonzalez-Cordero A, West EL, Donor and host photoreceptors engage in material transfer following transplantation of post-mitotic photoreceptor precursors. *Nat Commun*. 2016; 7: 13029. 27701378 10.1038/ncomms13029 PMC5059468

[35] Santos-Ferreira T, Llonch S, Borsch O, Postel K, Haas J, Ader M. Retinal transplantation of photoreceptors results in donor-host cytoplasmic exchange. *Nat Commun*. 2016; 7: 13028. 27701381 10.1038/ncomms13028 PMC5059459

[36] Singh MS, Balmer J, Barnard AR, Transplanted photoreceptor precursors transfer proteins to host photoreceptors by a mechanism of cytoplasmic fusion. *Nat Commun*. 2016; 7: 13537. 27901042 10.1038/ncomms13537 PMC5141374

[37] Francis PJ, Wang S, Zhang Y, Subretinal transplantation of forebrain progenitor cells in nonhuman primates: survival and intact retinal function. *Invest Ophthalmol Vis Sci*. 2009; 50: 3425–3431. 19234356 10.1167/iovs.08-2908 PMC2826708

[38] Klassen H, Kiilgaard JF, Warfvinge K, Photoreceptor differentiation following transplantation of allogeneic retinal progenitor cells to the dystrophic rhodopsin Pro347Leu transgenic pig. *Stem Cells Int*. 2012; 2012: 939801. 22567027 10.1155/2012/939801 PMC3337587

